# Systemic sclerosis sera affect fibrillin-1 deposition by dermal blood microvascular endothelial cells: therapeutic implications of cyclophosphamide

**DOI:** 10.1186/ar4270

**Published:** 2013-08-20

**Authors:** Marilisa Villano, Annalisa Borghini, Mirko Manetti, Erica Gabbrielli, Antonella Rossi, Piersante Sestini, Anna Franca Milia, Francesca Nacci, Serena Guiducci, Marco Matucci-Cerinic, Lidia Ibba-Manneschi, Elisabetta Weber

**Affiliations:** 1 Department of Molecular and Developmental Medicine, University of Siena, Via Aldo Moro 2, 53100, Siena, Italy; 2 Department of Experimental and Clinical Medicine, Section of Anatomy and Histology, University of Florence, Largo Brambilla 3, 50134, Florence, Italy; 3 Department of Medicine, Surgery and Neuroscience, University of Siena, Azienda Ospedaliera Universitaria Senese, Viale M Bracci 16, 53100, Siena, Italy; 4 Department of Experimental and Clinical Medicine, Section of Internal Medicine, Division of Rheumatology, University of Florence, Azienda Ospedaliero-Universitaria Careggi, Villa Monna Tessa, Viale G Pieraccini 18, 50139, Florence, Italy

**Keywords:** Systemic sclerosis, blood and lymphatic microvascular endothelial cells, fibrillin-1, focal adhesion molecules, cyclophosphamide

## Abstract

**Introduction:**

Systemic sclerosis (SSc) is a connective tissue disorder characterized by endothelial cell injury, autoimmunity and fibrosis. The following three fibrillin-1 alterations have been reported in SSc. (1) Fibrillin-1 microfibrils are disorganized in SSc dermis. (2) Fibrillin-1 microfibrils produced by SSc fibroblasts are unstable. (3) Mutations in the *FBN1 *gene and anti-fibrillin-1 autoantibodies have been reported in SSc. Fibrillin-1 microfibrils, which are abundantly produced by blood and lymphatic microvascular endothelial cells (B-MVECs and Ly-MVECs, respectively), sequester in the extracellular matrix the latent form of the potent profibrotic cytokine transforming growth factor β (TGF-β). In the present study, we evaluated the effects of SSc sera on the deposition of fibrillin-1 and microfibril-associated glycoprotein 1 (MAGP-1) and the expression of focal adhesion molecules by dermal B-MVECs and Ly-MVECs.

**Methods:**

Dermal B-MVECs and Ly-MVECs were challenged with sera from SSc patients who were treatment-naïve or under cyclophosphamide (CYC) treatment and with sera from healthy controls. Fibrillin-1/MAGP-1 synthesis and deposition and the expression of α_v_β_3 _integrin/phosphorylated focal adhesion kinase and vinculin/actin were evaluated by immunofluorescence and quantified by morphometric analysis.

**Results:**

Fibrillin-1 and MAGP-1 colocalized in all experimental conditions, forming a honeycomb pattern in B-MVECs and a dense mesh of short segments in Ly-MVECs. In B-MVECs, fibrillin-1/MAGP-1 production and α_v_β_3 _integrin expression significantly decreased upon challenge with sera from naïve SSc patients compared with healthy controls. Upon challenge of B-MVECs with sera from CYC-treated SSc patients, fibrillin-1/MAGP-1 and α_v_β_3 _integrin levels were comparable to those of cells treated with healthy sera. Ly-MVECs challenged with SSc sera did not differ from those treated with healthy control sera in the expression of any of the molecules assayed.

**Conclusions:**

Because of the critical role of fibrillin-1 in sequestering the latent form of TGF-β in the extracellular matrix, its decreased deposition by B-MVECs challenged with SSc sera might contribute to dermal fibrosis. In SSc, CYC treatment might limit fibrosis through the maintenance of physiologic fibrillin-1 synthesis and deposition by B-MVECs.

## Introduction

Fibrillins are large glycoproteins (approximately 350 kDa) present in the extracellular matrix (ECM). In lymphatic vessels, fibrillin microfibrils form filaments anchoring lymphatic endothelial cells (ECs) to the surrounding elastic fibers [1,2]. This structure modulates interstitial fluid entry into the lymphatic vessels, contributing to the physiologic tissue homeostasis [3]. Fibrillin microfibrils are also present in the arterial wall to maintain the elasticity which is usually lost in Marfan syndrome, where a fibrillin-1-encoding gene (*FBN1*) mutation fosters aortic dilatation and dissection [4,5]. During development, fibrillin constitutes a scaffold for elastin deposition. It also sequesters in the ECM signaling molecules, such as the profibrotic cytokine transforming growth factor β (TGF-β), which regulates ECM synthesis and remodeling [6]. Microfibril-associated glycoprotein 1 (MAGP-1) is a small glycoprotein of 31 kDa that resides on microfibril beads and colocalizes with virtually all fibrillin-1-containing microfibrils [7].

Fibrillin-1 is produced by different cell types [8-11], including blood and lymphatic ECs [12,13]. Its deposition differs in cultured bovine aortic and thoracic duct ECs. Blood ECs deposit fibrillin-1 in a honeycomb pattern with fibrillin-free spaces, whereas lymphatic ECs form a thick, irregular network of fibrillin-1 in the underlying ECM [12,13]. Blood and lymphatic microvascular ECs (hereafter referred to as B-MVECs and Ly-MVECs, respectively) isolated from human foreskin also deposit fibrillin-1 and MAGP-1 *in vitro *[14]. Fibrillin-1 microfibrils form a wide-mesh honeycomb, leaving fibrillin-free spaces that are gradually filled in by other microfibrils in both B-MVEC and Ly-MVEC cultures. In some Ly-MVECs, fibrillin-1 is initially deposited as uniformly scattered short fibrillin strands [14].

Alterations in fibrillin-1 have been reported in patients with systemic sclerosis (SSc, or scleroderma) and in the tight skin 1 (*Tsk1*) mouse model [15,16]. A disorganized deposition of fibrillin-1 has been observed in SSc skin [17]. Cultured dermal fibroblasts from SSc patients have been found to produce abnormal fibrillin-1 microfibrils which were unstable, less numerous and more susceptible to proteolytic degradation [18,19]. *Tsk1 *mice have a duplication in the *FBN1 *gene which results in a larger protein (450 kDa) that is more susceptible to proteolysis [20]. The instability of fibrillin-1 may cause an uncontrolled release of TGF-β, which is normally sequestered in the ECM as a latent inactive molecule, with consequent activation of fibroblasts and excessive collagen synthesis, ultimately leading to tissue fibrosis [21]. Moreover, microfibril fragments released in the ECM may reveal cryptic epitopes that may become the targets of an immune response with formation of anti-fibrillin-1 autoantibodies in SSc [22-24]. Associations of polymorphisms in the *FBN1 *gene and SSc susceptibility have also been reported in some ethnic groups with high prevalence of SSc (Choctaw American Indians and Japanese) [25-27].

It has been reported that latent TGF-β can be activated by interaction with α_v_β_3 _and α_v_β_5 _integrins that are overexpressed in cultured SSc dermal fibroblasts [28,29]. In particular, the α_v_β_3 _integrin may contribute to the establishment of an autocrine TGF-β loop potentiating fibrosis. Mutations in the arginine-glycine-aspartic acid (RGD) fibrillin-1 domain, which mediates integrin binding, have been found in the stiff skin syndrome, an autosomal dominant congenital form of scleroderma [30]. In stiff skin syndrome, altered cell-matrix interactions parallel an excessive microfibrillar deposition, altered elastogenesis and increased TGF-β signaling in the dermis.

Because the endothelium is one of the principal targets of SSc, we focused our attention on two physiologic activities of ECs that may be of relevance in this disease: fibrillin-1 deposition and focal adhesion molecule expression. We therefore evaluated the effect of sera from SSc patients on the deposition of fibrillin-1 and MAGP-1 and the expression of focal adhesion molecules by human adult dermal B-MVECs and Ly-MVECs. We also investigated the effect of the powerful immunosuppressant cyclophosphamide (CYC) on the deposition of fibrillin-1 and the expression of focal adhesion molecules by dermal B-MVECs and Ly-MVECs.

## Materials and methods

### Ethical approval

All SSc patients and control subjects signed an informed consent form. The study was conducted in compliance with the principles of the Declaration of Helsinki and was approved by the local institutional review board at the Azienda Ospedaliero-Universitaria Careggi, Florence, Italy.

### Patients and serum samples

Serum samples were obtained from a total of 21 consecutive patients with SSc (17 women, 4 men) recruited from the Division of Rheumatology, University of Florence. Patients with symptoms overlapping with those of other autoimmune, rheumatic and/or connective tissue diseases were excluded from the study. Eight age- and sex-matched healthy individuals were used as controls. Patients were classified as having limited cutaneous SSc (lSSc; *n *= 13) or diffuse cutaneous SSc (dSSc; *n *= 8) according to the categorization described by LeRoy *et al. *[31]. All SSc patients were clinically assessed as previously described [32]. Thirteen patients (eight with lSSc and five with dSSc) were receiving monthly intravenous infusions of CYC (dose range, 1 to 1.5 g/m^2 ^for 12 to 18 months), and the other eight patients were not taking any immunosuppressant or disease-modifying drugs. Blood was drawn from CYC-treated patients 1 month after the previous infusion. The demographic, clinical and serological characteristics of SSc patients are reported in Table 1. Fresh venous blood samples from patients and healthy controls were allowed to clot for 30 min before centrifugation at 1,500 *g *for 15 min. Serum was collected and stored in aliquots at -80°C until used.

**Table 1:** Demographic and clinical characteristics of the 21 patients with systemic sclerosis^a^

Characteristics	SSc patients (*N *= 21)
Mean age (±SD), yr	56.5 ± 12.1
Sex, *n *(%)	
Male	4 (19.1)
Female	17 (80.9)
Disease subset, *n *(%)	
lSSc	13 (61.9)
dSSc	8 (38.1)
Mean disease duration (±SD), yr^b^	7.7 ± 4.1
Autoantibody positivity, *n *(%)	
ANA	21 (100)
Anti-Scl-70	7 (33.3)
ACA	10 (47.6)
Digital ulcers, *n *(%)	12 (57.1)
Nailfold capillaroscopy pattern, *n *(%)	
Early	4 (19.0)
Active	8 (38.1)
Late	9 (42.9)
Mean skin score (±SD)	10.2 ± 6.3
ILD, *n *(%)^c^	11 (52.4)
CYC treatment, *n *(%)	13 (61.9)^d^

^a^ACA, anticentromere antibodies; ANA, antinuclear antibodies; Anti-Scl-70, anti-Scl-70 antibodies; CYC, cyclophosphamide; dSSc, diffuse cutaneous systemic sclerosis; ILD, interstitial lung disease; lSSc, limited cutaneous systemic sclerosis; SSc, systemic sclerosis. Except where indicated otherwise, values are the number (%) of participants. ^b^Disease duration was calculated from the date of the first non-Raynaud's symptom of SSc. ^c^Determined by high-resolution computed tomography scan. ^d^Eight patients with lSSc and five patients with dSSc.

### Cells

Human adult dermal B-MVECs and Ly-MVECs were obtained from Lonza (HMVEC-dBlAd and HMVEC-dLyAd, respectively; Lonza, Milan, Italy). Cells were seeded into 75-cm^2 ^flasks in complete Endothelial Growth Medium 2 (EGM-2) supplemented with the EGM-2-MV BulletKit (Lonza). Once at confluence, cells were trypsinized with a trypsin/ethylenediaminetetraacetic acid solution (Lonza), centrifuged, resuspended in medium with EGM-2-MV and seeded onto gelatin-coated coverslips at a density of 4.5 × 10^4 ^cells/coverslip. At confluence, all medium was removed and cells were starved in endothelial basal medium (EBM) for 2 h, then fed with fresh EBM containing 15% of serum from lSSc or dSSc patients, naïve or under pharmacological therapy with CYC, or from healthy controls. Fibrillin-1 evaluation was performed on cells at 2 days after confluence because it has been shown that fibrillin-1 deposition *in vitro *increases with time in culture [33]. The expression of focal adhesion molecules and the actin cytoskeleton assembly were also evaluated at the same time point.

### Double-immunolabeling of fibrillin-1 and microfibril-associated glycoprotein 1

After fixation in cold acetone for 7 min, cells were washed in phosphate-buffered saline (PBS) and incubated for 40 min in 3% bovine serum albumin (BSA) in PBS to block unspecific binding sites. The first immunolabeling was performed by overnight incubation at 4°C with a polyclonal antibody to MAGP-1 (MFAP-2; Sigma-Aldrich, Milan, Italy) diluted 1:25 in PBS containing 0.5% BSA followed by incubation for 1 h with a goat anti-rabbit Alexa Fluor 594-conjugated immunoglobulin G (IgG) antibody (1:1,000 dilution in PBS with 1% BSA; Invitrogen, Milan, Italy). Before the second immunolabeling, unspecific binding sites were again blocked. Cells were then incubated for 2 h with a monoclonal antibody to fibrillin-1 (1:100 dilution in PBS with 0.5% BSA; Chemicon International/Millipore, Milan, Italy). The reaction was revealed by incubation for 1 h with goat anti-mouse Alexa Fluor 488-conjugated IgG1 (1:1,000 dilution in PBS with 1% BSA; Invitrogen). Nuclei were counterstained for 10 min with 4′,6-diamidino-2-phenylindole (DAPI, 1:1,000 dilution in PBS; Sigma-Aldrich). After being washed, coverslips were mounted on glass slides with DABCO mounting medium (Sigma-Aldrich).

### Double-immunolabeling of adhesion molecules

Cells were fixed for 7 min in 4% buffered paraformaldehyde, permeabilized for 10 min with 0.1% Triton X-100 in PBS, washed, and incubated for 30 min in PBS with 1% BSA to block unspecific binding sites. The latter buffer was used for all subsequent dilutions. For double-labeling with α_v_β_3 _integrin and phosphorylated focal adhesion kinase (FAK), the first labeling was performed by overnight incubation at 4°C with a mouse monoclonal antibody to human α_v_β_3 _integrin (vitronectin receptor, 1:50 dilution; Chemicon International), followed by incubation for 1 h with a goat anti-mouse antibody, Alexa Fluor 488-conjugated IgG (1:1,000 dilution; Invitrogen). After unspecific binding sites were blocked, cells were washed and incubated for 2 h with a rabbit polyclonal antibody to phosphorylated FAK (anti-FAK (phospho Y397), 1:100 dilution; Abcam, Cambridge, UK). For double-labeling with phalloidin and vinculin, the first labeling was performed with a 20-min incubation with Alexa 488 phalloidin (1:40 dilution; Invitrogen), followed by block of unspecific binding sites and then by overnight incubation with a monoclonal antibody to vinculin (1:100 dilution; Sigma-Aldrich). The reaction was revealed by 1-h incubation with goat anti-mouse Alexa Fluor 594-conjugated IgG1 (1:1,000 dilution; Invitrogen). Cell nuclei were counterstained with DAPI, and coverslips were mounted with DABCO mounting medium.

### Morphometric analysis

Coverslips with adhering cells were photographed in randomly selected fields under a ×40 objective while maintaining fixed exposure parameters. Fluorescence was analyzed using the NIS-Elements D morphometric software program (Nikon, Tokyo, Japan) on at least 35 photomicrographs/condition for fibrillin-1 and MAGP-1 and 15 photomicrographs/condition for focal adhesion molecules. The amount of fluorescence was expressed as sum density, which is the sum of individual optical density (O.D.) of each pixel in the area being measured. O.D. is evaluated according to the following formula: O.D. = -log((pixel intensity value + 0.5)/62.5).

### Statistical analysis

Data presented are means and standard errors of the mean (SEM). Statistical analysis was performed using Student's *t*-test for independent groups. A *P*-value less than 0.001 according to a two-tailed distribution was considered statistically significant unless otherwise specified.

## Results

### Morphologic evaluation of fibrillin-1 and microfibril-associated glycoprotein 1 deposition by dermal B-MVECs and Ly-MVECs

**Figure 1:**
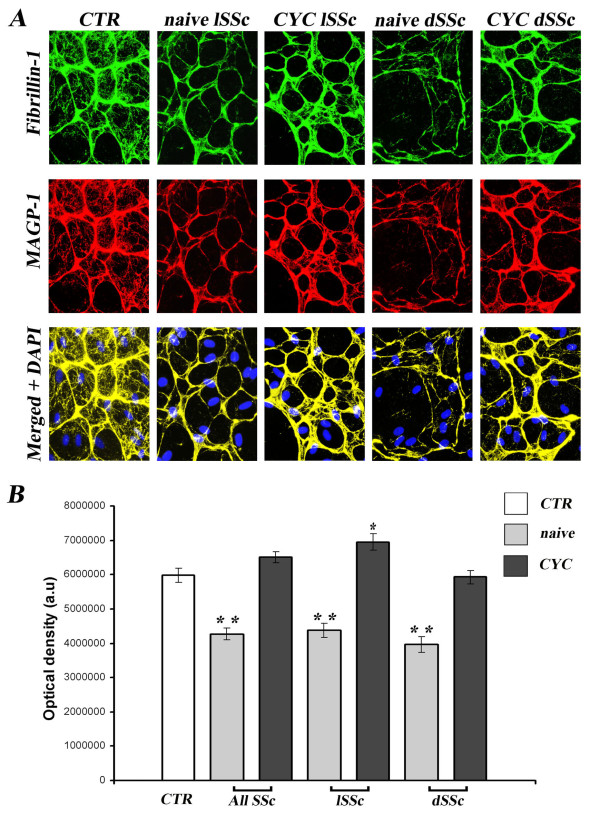
**Systemic sclerosis sera affect deposition of fibrillin-1 and microfibril-associated glycoprotein 1 by dermal blood microvascular endothelial cells**. Fibrillin-1 and microfibril-associated glycoprotein 1 (MAGP-1) deposition by dermal blood microvascular endothelial cells (B-MVECs) was evaluated after challenge with systemic sclerosis (SSc) and healthy control (CTR) sera. **(A) **Representative immunofluorescent images for fibrillin-1 (green) and MAGP-1 (red). The two molecules colocalize and are deposited in a honeycomb pattern, with empty spaces in which endothelial cells are still visible. 4′,6-diamidino-2-phenylindole (DAPI)-stained nuclei are blue in merged images. Original magnification, ×40. **(B) **Quantification of fibrillin-1 deposition by measurement of optical density in arbitrary units (a.u.). Data are means ± SEM. Fibrillin-1 deposition was significantly reduced in B-MVECs challenged with naïve SSc sera (all naïve SSc, naïve limited SSc (lSSc) and naïve diffuse SSc (dSSc)) vs. healthy controls (**P < 0.001). Fibrillin-1 was significantly higher in cyclophosphamide (CYC)-treated lSSc than in controls (*P < 0.05). No significant difference was found between all CYC-treated SSc and CYC-treated dSSc vs. healthy controls.

**Figure 2:**
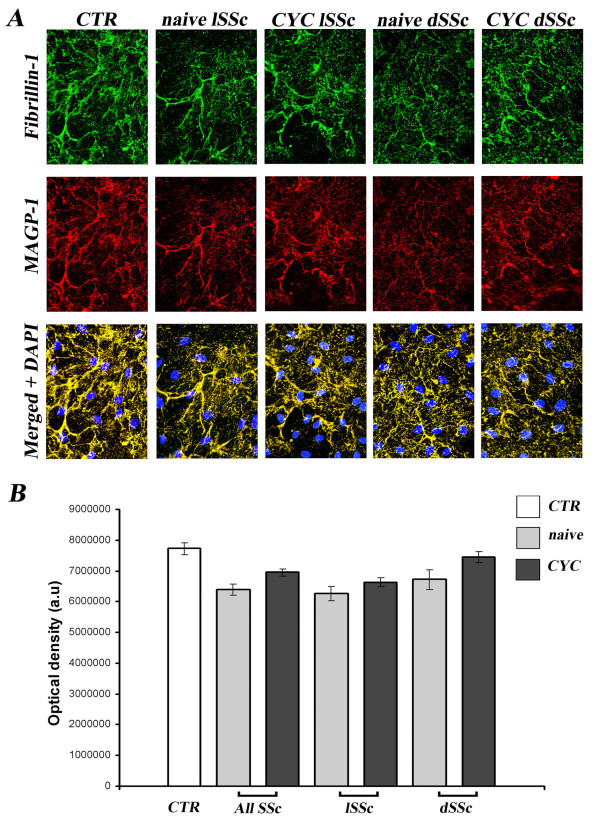
**Lack of effect of systemic sclerosis sera on deposition of fibrillin-1 and microfibril-associated glycoprotein 1 by dermal lymphatic microvascular endothelial cells**. Fibrillin-1 and microfibril-associated glycoprotein 1 (MAGP-1) deposition by dermal lymphatic microvascular endothelial cells (Ly-MVECs) was evaluated after challenge with systemic sclerosis (SSc) and healthy control (CTR) sera. **(A) **Representative immunofluorescent images for fibrillin-1 (green) and MAGP-1 (red). The two molecules colocalize and are deposited in thin, short strands, only rarely forming honeycombs. 4′,6-diamidino-2-phenylindole (DAPI)-stained nuclei are blue in merged images. Original magnification, ×40. **(B) **Quantification of fibrillin-1 deposition by measurement of optical density in arbitrary units (a.u.). Data are means ± SEM. No significant differences vs. healthy controls were found in any of the conditions assayed. CYC, cyclophosphamide; dSSc, diffuse SSc; lSSc, limited SSc.

Fibrillin-1 and MAGP-1 colocalized in dermal B-MVECs and Ly-MVECs in all experimental conditions tested (Figures 1A and 2A) in accordance with previous reports in the literature [7] and our previous results in human foreskin microvascular ECs [14]. The deposition pattern of the two proteins differed between B-MVECs and Ly-MVECs, however. In B-MVECs, fibrillin-1 microfibrils formed a wide mesh with a honeycomb pattern with fibrillin-1-free spaces in which cells were still visible (Figure 1A). Thin strands of fibrillin-1 arising from the honeycomb borders subdivided fibrillin-1-free spaces into smaller ones, and the space between different honeycombs was also filled by a thin network of fibrillin-1 fibers. In Ly-MVECs, fibrillin-1 microfibrils were deposited as short, thin strands forming a thick mesh covering the surface (Figure 2A). Honeycombs were rarely observed in Ly-MVEC cultures.

### Quantitative analysis of fibrillin-1 and microfibril-associated glycoprotein 1 deposition by dermal B-MVECs and Ly-MVECs challenged with systemic sclerosis sera

In B-MVECs, the production and deposition of fibrillin-1 and MAGP-1 were significantly lower in cultures challenged with sera from naïve SSc patients than in cultures challenged with healthy control sera (*P *< 0.001) (Figure 1B). No significant differences were observed between cells challenged with lSSc and dSSc naïve sera (Figure 1B). Upon challenge with sera from CYC-treated SSc patients, B-MVECs deposited fibrillin-1/MAGP-1 at levels comparable to those of cells treated with healthy control sera (Figure 1B). In particular, no significant difference in fibrillin-1/MAGP-1 deposition was found between B-MVECs stimulated with sera from healthy controls and those stimulated with CYC-treated dSSc sera (Figure 1B). Fibrillin-1/MAGP-1 values upon challenge with CYC-treated lSSc sera were even higher (*P *< 0.05) than those of healthy controls (Figure 1B). In Ly-MVECs, there were no significant differences between cultures challenged with sera from healthy controls, naïve SSc patients and CYC-treated SSc patients (Figure 2B).

### Quantitative analysis of α_v_β_3 _integrin, phosphorylated FAK, vinculin and actin expression by dermal B-MVECs and Ly-MVECs challenged with systemic sclerosis sera

**Figure 3:**
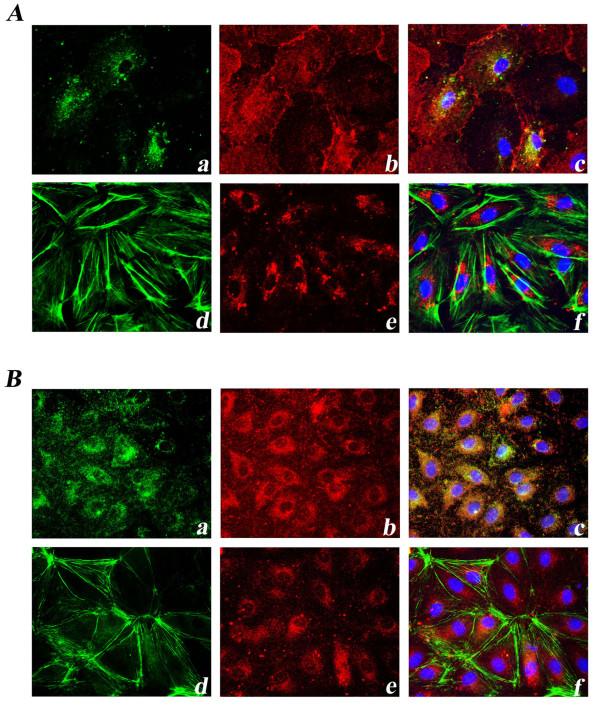
**Expression of focal adhesion molecules in dermal blood microvascular endothelial cells and lymphatic microvascular endothelial cells**. Representative immunofluorescent images of focal adhesion molecules in blood microvascular endothelial cells (B-MVECs) **(A) **and lymphatic microvascular endothelial cells (Ly-MVECs) **(B) **cultured with healthy sera are shown. Representative immunofluorescent photomicrographs show double-labeling for α_v_β_3 _integrin (green) **(a)**, phosphorylated focal adhesion kinase (FAK) (red) **(b) **and a merged image **(c)**. Representative immunofluorescent photomicrographs show double-labeling for phalloidin (green) **(d)**, vinculin (red) **(e) **and a merged image **(f)**. 4′,6-diamidino-2-phenylindole (DAPI)-stained nuclei are blue in merged images. In both B-MVECs and Ly-MVECs, α_v_β_3 _integrin (a) and phosphorylated FAK (b) are co-localized. α_v_β_3 _integrin (a), phosphorylated FAK (b) and vinculin (e) are visible as small dots distributed all over the cell surface. Phalloidin-stained actin filaments (d) are arranged in long strands running parallel to cell borders. Original magnification, ×40.

Both in B-MVECs and Ly-MVECs, α_v_β_3 _integrin, phosphorylated FAK and vinculin were visible as small dots distributed all over the cell surface. Actin filaments stained by phalloidin were principally arranged in long strands running parallel to cell borders (Figure 3).

**Figure 4:**
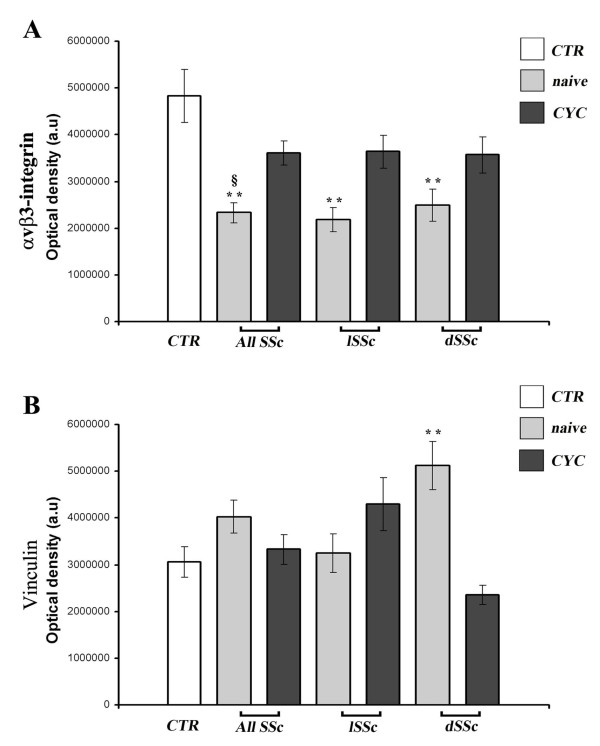
**Systemic sclerosis sera affect α_v_β_3 _integrin and vinculin expression in dermal blood microvascular endothelial cells**. Quantification of α_v_β_3 _integrin **(A) **and vinculin **(B) **in dermal blood microvascular endothelial cells (B-MVECs) challenged with systemic sclerosis (SSc) and healthy control (CTR) sera. Data are means ± SEM of optical density in arbitrary units (a.u.). **(A) **The expression of α_v_β_3 _integrin was significantly lower in cells challenged with sera from naïve SSc patients (all naïve SSc, naïve limited SSc (lSSc) and naïve diffuse SSc (dSSc)) than in cells treated with sera from healthy controls (**P < 0.001). No significant differences were found between cyclophosphamide (CYC)-treated SSc patients (all SSc, lSSc and dSSc) and healthy controls, whereas expression of α_v_β_3 _integrin in all CYC-treated SSc was significantly higher than that in all naïve SSc (^§^*P *< 0.001). **(B) **The expression of vinculin was significantly higher in cells challenged with the sera of naïve dSSc than in cells treated with sera from healthy controls (**P < 0.001). No significant differences were found in any of the other conditions tested.

In B-MVECs, the expression of α_v_β_3 _integrin was significantly lower in cells challenged with the serum of naïve SSc patients than in healthy controls (*P *< 0.001) (Figure 4A). No significant differences were observed between cells challenged with lSSc and dSSc naïve sera (Figure 4A). Upon challenging of B-MVECs with sera from CYC-treated SSc patients, the levels of α_v_β_3 _integrin expression were similar to those in cells treated with healthy control sera (Figure 4A). Moreover, the expression of α_v_β_3 _integrin was higher in cells challenged with sera of CYC-treated SSc patients compared with treatment-naïve SSc patients (*P *< 0.001) (Figure 4A).

Vinculin expression was significantly higher in B-MVECs challenged with sera of treatment-naïve dSSc patients than in sera of healthy controls (*P *< 0.001) (Figure 4B). We failed to detect any significant difference in phosphorylated FAK and actin expression among the different experimental conditions (data not shown). In Ly-MVECs, no significant differences could be detected for any of the focal adhesion molecules tested among the different experimental conditions (data not shown).

## Discussion

Our work shows for the first time that a reduced deposition of fibrillin-1 and dysregulated expression of focal adhesion molecules by dermal B-MVECs may have an important role in SSc pathophysiology, and that the beneficial effects of CYC treatment may be due in part to the normalization of cell-matrix interactions and fibrillin-1 deposition by B-MVECs.

In the literature, there are controversial data on fibrillin deposition in SSc. In the lower dermis of localized scleroderma and SSc, Fleischmajer *et al. *[17] described a massive deposition of 10-nm microfibrils interspersed among collagen fibers. They suggested that the increase in fibrillin deposition in the ECM might be the result of an abnormal activation of dermal fibroblasts that leads to significant accumulation of collagen and microfibrils. Other authors have reported a decrease of the microfibrillar network in biopsies from both clinically affected and unaffected dSSc skin [36]. Data on fibrillin deposition by cultured SSc dermal fibroblasts are also controversial. Some authors have found an increased production of fibrillin-1 in SSc fibroblasts [34,35], but other authors have found no difference in the expression of fibrillin-1 between SSc fibroblasts from involved and uninvolved skin and controls [36]. Another *in vitro *study reported a reduction in fibrillin deposition by SSc fibroblasts from involved skin [18].

In the present study, we investigated for the first time the possible contribution of dermal microvascular ECs to fibrillin deposition abnormalities in SSc. It is well-known that blood and lymphatic ECs synthesize and deposit fibrillin-1 in the ECM and that the endothelium is one of the earliest and most important targets in SSc pathogenesis [12-14,37]. In particular, we used dermal B-MVECs and Ly-MVECs obtained from healthy individuals and challenged them with SSc sera. The rationale for investigating both B-MVECs and Ly-MVECs comes from the recent findings that not only blood microvessels but also lymphatic microvessels are affected in SSc [38-40]. Moreover, an early involvement of lymphatic vessels in SSc may be suggested by the presence of puffy fingers, which often precedes the onset of fibrosis [41].

Our data on significant reduction of fibrillin-1 deposition by B-MVECs cultured with SSc sera are in agreement with previous findings on reduced fibrillin-1 in SSc skin biopsies and fibroblast cultures [18,36]. The present findings were revealed by using sera from both lSSc and dSSc patients who were not under treatment with immunosuppressant or disease-modifying drugs. The fact that stimulation of B-MVECs with sera from CYC-treated SSc maintained normal fibrillin-1 deposition may suggest that the immunosuppressive effect of CYC has a role in redirection, through unknown mechanisms, of the deposition of fibrillin-1 in the ECM. It has been reported that anti-fibrillin-1 antibodies are present in about 34% to 80% of SSc patients and can exert profibrotic functions through TGF-β-mediated mechanisms [21]. In the ECM, these autoantibodies may interfere with the fibrillin-1-mediated stabilization of latent TGF-β. Interestingly, CYC exerts its anti-inflammatory and immunosuppressive functions through direct cytoxicity of bone marrow precursors and mature lymphocytes, with consequent reduction of B and T cells [42]. Therefore, our findings might be explained by the possible CYC-mediated reduction in circulating levels of anti-fibrillin-1 autoantibodies and/or T-cell-derived proinflammatory molecules active on the endothelium. However, we did not test the presence of such autoantibodies or proinflammatory cytokines in our SSc serum samples. Further studies using specific blocking agents (for example, anti-TGF-β antibodies or TGF-β receptor inhibitors) or heat-inactivated serum samples will help to provide a mechanistic explanation for the differential serum effects reported herein. Certainly, the observed effects of CYC in our experimental conditions are not due to EC proliferation, because fibrillin-1 deposition was evaluated in postconfluent, quiescent EC cultures. Because of the critical role of fibrillin-1 in sequestering latent TGF-β in the ECM and in stabilizing blood vessel wall [6], a normal deposition of fibrillin-1 by B-MVECs, as observed upon challenge with CYC-treated SSc sera, might contribute either indirectly to the control of fibroblast activation, hence limiting fibrosis, or directly to microvascular deremodeling. In the present study, we focused on healthy dermal MVECs challenged with SSc sera. Further research is required to investigate fibrillin-1 deposition by MVECs isolated from the affected dermis of SSc patients. Indeed, we cannot exclude the possibility that severely damaged dermal MVECs, such as those of SSc patients, might be less responsive to the beneficial effect of CYC, thus highlighting the necessity of an early diagnosis and start of treatment.

We also investigated focal adhesion molecules and observed a decreased α_v_β_3 _integrin expression in B-MVECs challenged with sera from treatment-naïve SSc patients (both lSSc and dSSc). The discrepancy between our results and those of other studies showing an overexpression of α_v_β_3 _integrins in fibroblasts isolated from affected SSc skin may be due to the different cell types employed (ECs vs. fibroblasts) [28]. In the context of SSc, an overexpression of α_v_β_3 _integrin in fibroblasts, leading to increased production of collagen, may be regarded as a negative event. Similarly, the observed decreased levels of EC α_v_β_3 _integrin expression may also be interpreted as a negative event because integrins are necessary for the normal interaction of ECs with ECM proteins, particularly fibrillin, and hence for fibrillin microfibril stabilization [43]. As observed for fibrillin-1 deposition, the stimulation of B-MVECs with CYC-treated SSc sera showed a potentially beneficial effect by maintaining normal integrin α_v_β_3 _expression. Integrins are essential in triggering FAK phosphorylation, which in turn initiates a cascade that eventually leads to cell adaptation to environmental stimuli [12]. However, the decrease in α_v_β_3 _integrin expression following stimulation with naïve SSc sera, which we report herein, was not associated with differences in FAK phosphorylation. Other integrins present on ECs, such as α_5_β_1 _integrin, may account for α_v_β_3_-independent activation of FAK [44]. Moreover, several molecules that signal through G protein-coupled receptors, including neuropeptides, oncogenes, hormones and growth factors, present in patient's serum might trigger integrin-independent FAK phosphorylation [45,46].

We also observed increased vinculin expression in B-MVECs challenged with sera from treatment-naïve dSSc patients. This finding is in line with the previously reported TGF-β-mediated increased production of vinculin-containing adhesion complexes in SSc myofibroblasts [47]. The increased vinculin-mediated adhesion to the ECM has been proposed as a protective factor against myofibroblast apoptosis, hence ultimately contributing to fibrosis [47]. Because timely modulation of expression and assembly of endothelial vinculin are crucial during angiogenesis [48], dysregulated expression of vinculin might have a role in the impaired angiogenic process found in SSc [37]. The stimulation of B-MVECs with sera from CYC-treated dSSc patients showed a potentially beneficial effect by maintaining vinculin expression at normal levels. This hypothesis is in agreement with the previous evidence that CYC is able to partially deremodel the damaged microvasculature in SSc, as observed by nailfold videocapillaroscopy [49]. The observed significant increase in vinculin expression in B-MVECs challenged with naïve dSSc sera, but not in cells stimulated with lSSc sera, might be explained in part by the fact that vinculin is one of the main proteins affected by connective tissue growth factor [50], whose serum levels are increased, especially in patients with the diffuse form of the disease [51]. In contrast to the findings obtained with B-MVECs, Ly-MVECs challenged with SSc sera did not differ from healthy controls in the expression of fibrillin-1 and any of the adhesion molecules assayed. This difference may be due to the fact that, under *in vivo *conditions, the lymphatic endothelium is never directly exposed to the as yet undiscovered serum factors that seem to affect the relationship between B-MVECs and the surrounding ECM.

## Conclusions

Our data clearly point to a possible role for a dysregulated deposition of fibrillin-1 and expression of focal adhesion molecules by dermal B-MVECs in SSc pathophysiology. Moreover, for the first time, we have shown that the beneficial effects of CYC treatment in SSc may be due in part to the normalization of cell-matrix interactions and fibrillin-1 deposition by B-MVECs.

## Abbreviations

B-MVEC: blood microvascular endothelial cell; BSA: bovine serum albumin; CYC: cyclophosphamide; dSSc: diffuse cutaneous systemic sclerosis; EBM: endothelial basal medium; EC: endothelial cell; ECM: extracellular matrix; EGM-2: endothelial growth medium 2; IgG: immunoglobulin G; Ly-MVEC: lymphatic microvascular endothelial cell; lSSc: limited cutaneous systemic sclerosis; MAGP-1: microfibril-associated glycoprotein 1; O.D.: optical density; PBS: phosphate-buffered saline; SSc: systemic sclerosis; TGF-β: transforming growth factor β

## Competing interests

The authors declare that they have no competing interests.

## Authors' contributions

All authors meet the criteria for authorship. MV, EG and AR contributed to the *in vitro *assays. AB contributed to the *in vitro *assays and data analysis. MM participated in study design and coordination and contributed to data analysis and interpretation. PS and AFM contributed to data analysis and interpretation. FN and SG collected and supplied serum samples and clinical data. MMC, LIM and EW conceived the study and participated in its design and coordination. All authors contributed to the drafting and editing of the manuscript. All authors read and approved the final manuscript for publication.
